# Frequency of dysplasia in endoscopically resected pseudopolyps in inflammatory bowel diseases

**DOI:** 10.1093/ecco-jcc/jjaf196

**Published:** 2025-11-19

**Authors:** Elena De Cristofaro, Irene Marafini, Martina Franchin, Chiara Venuto, Luca Savino, Elisabetta Lolli, Giorgia Sena, Benedetto Neri, Francesca Zorzi, Edoardo Troncone, Livia Biancone, Giovanna Del Vecchio Blanco, Augusto Orlandi, Emma Calabrese, Giovanni Monteleone

**Affiliations:** Gastroenterology Unit, Policlinico Universitario “Tor Vergata,” Rome, Italy; Gastroenterology Unit, Policlinico Universitario “Tor Vergata,” Rome, Italy; Department of Systems Medicine, University of “Tor Vergata,” Rome, Italy; Gastroenterology Unit, Policlinico Universitario “Tor Vergata,” Rome, Italy; Gastroenterology Unit, Policlinico Universitario “Tor Vergata,” Rome, Italy; Pathology Unit, Policlinico Universitario “Tor Vergata,” Rome, Italy; Gastroenterology Unit, Policlinico Universitario “Tor Vergata,” Rome, Italy; Gastroenterology Unit, Policlinico Universitario “Tor Vergata,” Rome, Italy; Therapeutic GI Endoscopy Unit, Fondazione Policlinico Universitario Campus Bio-Medico, Rome, Italy; Gastroenterology Unit, Policlinico Universitario “Tor Vergata,” Rome, Italy; Gastroenterology Unit, Policlinico Universitario “Tor Vergata,” Rome, Italy; Gastroenterology Unit, Policlinico Universitario “Tor Vergata,” Rome, Italy; Department of Systems Medicine, University of “Tor Vergata,” Rome, Italy; Gastroenterology Unit, Policlinico Universitario “Tor Vergata,” Rome, Italy; Department of Systems Medicine, University of “Tor Vergata,” Rome, Italy; Pathology Unit, Policlinico Universitario “Tor Vergata,” Rome, Italy; Gastroenterology Unit, Policlinico Universitario “Tor Vergata,” Rome, Italy; Department of Systems Medicine, University of “Tor Vergata,” Rome, Italy; Gastroenterology Unit, Policlinico Universitario “Tor Vergata,” Rome, Italy; Department of Systems Medicine, University of “Tor Vergata,” Rome, Italy

**Keywords:** post-inflammatory polyps, inflammatory bowel disease, pseudopolyps, dysplasia

## Abstract

**Background and Aims:**

Pseudopolyps have traditionally been considered sequelae of mucosal healing in inflammatory bowel diseases (IBDs). However, recent retrospective studies suggest that pseudopolyps may harbor dysplasia or obscure neoplastic lesions. This prospective study aimed to assess the frequency of dysplasia in pseudopolypoid lesions endoscopically resected in IBD patients and to identify potential predictors of dysplasia.

**Methods:**

We analyzed pseudopolypoid lesions resected during colonoscopies performed between June 2023 and March 2025 in patients with colonic IBD at a single tertiary center. Lesions macroscopically classified as pseudopolyps and completely resected were histologically analyzed and categorized as inflammatory pseudopolyps, inflammatory pseudopolyps with foci of epithelial dysplasia, conventional adenomas, or IBD-associated dysplasia. Multivariable logistic regression was used to identify predictors of dysplasia.

**Results:**

Pseudopolyps were identified in 165 out of 910 patients undergoing colonoscopy (18.1%), and 124 lesions were resected in 98 patients. Dysplasia was detected in 15 lesions (12.1%), including conventional adenomas (53%, one with intramucosal carcinoma/high-grade dysplasia), IBD-associated dysplasia (20%), and hyperplastic lesions with dysplasia (27%). A heterogeneous pit pattern (OR = 4.50; 95% CI: 1.27-15.9) and absence of ulceration (OR = 0.093; 95% CI: 0.01-0.77) were independent predictors of dysplasia. No dysplasia was found in the surrounding mucosa.

**Conclusions:**

Dysplasia was found in 12% of pseudopolypoid lesions, challenging the assumption that they are uniformly benign. Endoscopic features such as heterogeneous pit pattern and absence of ulceration may aid in identifying high-risk lesions. These results highlight the diagnostic uncertainty surrounding pseudopolyps in IBD and call for careful endoscopic assessment rather than routine resection.

## 1. Introduction

Inflammatory bowel diseases (IBDs), including Crohn’s disease (CD) and ulcerative colitis (UC), are chronic relapsing inflammatory conditions of the gastrointestinal tract associated with an increased risk of colorectal cancer (CRC),^1^^,^^2^ which accounts for up to 10% of IBD-related deaths despite the use of drugs that have the potential to interfere with colon carcinogenesis.^3^^,^^4^ Current surveillance strategies focus on the early detection of colitis-associated neoplasia through periodic colonoscopies, especially in patients with long-standing and extensive colitis.^5^

Pseudopolyps, also known as post-inflammatory polyps, are common findings in patients with IBD, particularly in those with extensive or chronically active disease. These lesions arise due to regeneration and re-epithelialization during the healing phase of the inflammatory process.^6–8^ Although historically considered benign, pseudopolyps have been proposed as a potential marker of CRC risk. However, this association has not been consistently confirmed across studies.^9–14^ The potential neoplastic risk associated with pseudopolyps may stem from the possibility that, when particularly numerous, pseudopolyps could obscure dysplastic lesions during surveillance. Additionally, some subsets of pseudopolyps may harbor dysplastic changes.^15–18^ Currently, routine resection of pseudopolyps is not generally recommended unless they exhibit suspicious features, such as mucosal irregularity or atypical characteristics suggestive of dysplasia, including changes in color or vascular pattern, focal nodularity, elevation, or ulceration, which may interfere with pit pattern assessment. However, the endoscopic evaluation of pseudopolyps can be challenging, as inflammation may distort pit patterns independently of the presence of dysplasia. Indeed, image-enhanced endoscopy and advanced diagnostic technologies have yielded conflicting results in distinguishing between inflammatory and dysplastic polypoid lesions in IBD patients.^13^^,^^19–24^ Commonly used classification systems, such as the Kudo and JNET classifications, are not routinely applied to IBD-related lesions due to their limited accuracy in distinguishing neoplastic from non-neoplastic changes in this context. The modified Kudo classification, combined with FICE, has shown improved accuracy compared to white-light endoscopy in UC surveillance.^25^ However, the interpretation of pit and vascular patterns remains challenging in areas of chronic inflammation, particularly within pseudopolypoid mucosa.^19^

Despite these limitations, no previous study has systematically evaluated the true frequency of dysplasia in pseudopolypoid lesions that were completely resected endoscopically. In routine clinical practice, such lesions are often sampled with a single biopsy, which may not fully represent the entire lesion and could miss focal areas of dysplasia. In our recent retrospective study, dysplasia was identified in approximately 25% of pseudopolyps biopsied or resected,^15^ suggesting that pseudopolyps themselves may occasionally represent or mask true neoplastic lesions.

This prospective study aimed to evaluate the prevalence of dysplasia in lesions with a pseudopolypoid appearance that were endoscopically resected in IBD patients, and to identify potential predictors of dysplasia.

## 2. Methods

### 2.1. Study design and population

We prospectively analyzed IBD data from a single-center study at Tor Vergata University Hospital (Rome, Italy), including patients with colonic IBD who underwent endoscopic resection of pseudopolypoid lesions between June 2023 and March 2025. The procedure was either performed during surveillance or clinically indicated colonoscopies by three expert endoscopists each with extensive experience (performing >1000 colonoscopies with an adenoma detection rate ≥25% and at least 500 colonoscopies in IBD patients over more than 10 years). All the pseudopolypoid resections were performed at the discretion of the endoscopist, based on clinical judgment. Patients with pseudopolypoid lesions that were described but not resected were excluded. Reasons for non-resection were documented and included ongoing anticoagulant or antiplatelet therapy, lack of dedicated time during a diagnostic colonoscopy, patient intolerance, and inadequate bowel preparation (defined as a total Boston Bowel Preparation Scale score <6 or any segmental score ≤1).

Additional exclusion criteria were incomplete colonoscopy (defined as failure to reach the cecum) and the presence of numerous pseudopolyps that impaired visualization.

Disease activity was assessed using the Harvey Bradshaw Index (HBI) for CD and the Partial Mayo Score for UC. Ongoing treatments were documented, along with any optimization or therapy changes. Additionally, clinical history was collected, including smoking habits, CRC family history, previous dysplasia findings, prior use of biologic and/or immunosuppressive therapies, and history of related IBD surgery. The study was approved by the local Ethics Committee (N. 2022/106.22).

### 2.2. Procedures

Colonoscopy procedures were performed using high-definition endoscopes (CFHQ1100, CFH190, CFH185; Olympus Medical Systems Corporation, Tokyo, Japan) according to clinical practice. In patients with long-standing disease, colonoscopy was performed using either dye-based or virtual chromoendoscopy, with random biopsies taken when appropriate, in accordance with current surveillance recommendations. All patients underwent conscious sedation, sedoanalgesia, or deep sedation. Informed consent for colonoscopy, polypectomy, and sedation (conscious or deep) was obtained prior to the procedure. Cecal intubation was confirmed by identification of the appendiceal orifice and the ileocecal valve. A minimum withdrawal time of at least 6 min was required, extended to 20 min during chromoendoscopy to ensure thorough mucosal assessment.

Polyps endoscopically classified as post-inflammatory (eg, having hyperemic and friable mucosa, elongated contours, a “stuck-on” appearance, and absence of a discernible pit or vascular pattern)^19^ were also described based on their location, morphology (according to the Paris classification and classified as polypoid and non-polypoid), and the presence of surface ulceration. The number of pseudopolyps was recorded and classified as isolated (<10 pseudopolyps) or multiple. Glandular patterns were evaluated using white light, narrow-band imaging (NBI), and, when deemed necessary, dye-based chromoendoscopy with vital or contrast agents (methylene blue or indigo carmine). These patterns were described as either homogeneous when the surface architecture appeared macroscopically uniform in terms of glandular opening and structure throughout the lesion (Figure S1) or heterogeneous when the glandular pattern could not be consistently visualized or appeared irregular across different areas of the lesion.

The resection technique, polypectomy or endoscopic mucosal resection (EMR), either cold or hot, was selected by the endoscopist based on lesion characteristics, including location and size. Three biopsy samples were obtained from the adjacent mucosa to assess dysplasia. Endoscopic disease activity, assessed using the Simple Endoscopic Score for Crohn’s disease (SES-CD) for CD and the Mayo endoscopic score for UC, was also systematically evaluated and recorded in all patients.

### 2.3. Histological analysis

All resected lesions were fully embedded for histological analysis. In all samples, a minimum of three sections are routinely prepared at different levels. For cases with complex morphology or diagnostic uncertainty (eg, presence of dysplasia), additional deeper sections were obtained to ensure a comprehensive histological assessment. The resected polyps were histologically classified into four categories based on morphological criteria.^1^ Inflammatory pseudopolyps are characterized by a polypoid shape and increased inflammation, consisting of a dense mixture of lymphocytes, plasma cells, mast cells, neutrophils, and eosinophils infiltrating the intestinal lamina propria. These lesions can exhibit various degrees of surface erosion, crypt distortion or dilation, and hyperplasia, along with reactive nuclear changes in the mucosal epithelial cells. In some cases, samples may consist entirely of granulation tissue.^2^ Inflammatory pseudopolyps with foci of epithelial dysplasia;^3^ conventional colorectal adenoma is defined as a discrete lesion with clear signs of dysplasia, in the absence of inflammatory background in the surrounding mucosa;^4^ and IBD-associated dysplastic lesion is an unequivocal neoplastic alteration of the intestinal epithelium confined within the basement membrane, occurring in the context of IBD. All diagnoses were made by an expert gastrointestinal pathologist. In challenging cases, immunohistochemical staining for p53, Ki-67, and cytokeratin AE1/AE3 was performed, and additional hematoxylin and eosin sections were obtained at deeper levels. All slides with a diagnosis of dysplasia were subsequently reviewed and discussed by a second experienced gastrointestinal pathologist from our institution to confirm the diagnosis. Any discrepancies were resolved by consensus.

### 2.4. Outcomes

The primary outcome was to determine the frequency of dysplasia in colonic lesions that were macroscopically identified as pseudopolyps by the endoscopist. Secondary outcomes included identifying clinical and endoscopic factors associated with dysplasia, as well as evaluating the misclassification rate, defined as the proportion of lesions initially diagnosed endoscopically as pseudopolyps that were later found to be a different histological entity.

### 2.5. Statistical analysis

Continuous variables were assessed for normality using the Shapiro–Wilk test. For variables with a normal distribution, data were reported as mean and SD, while for non-normally distributed variables data were expressed as median and IQR. Categorical variables were reported as absolute numbers and percentages.

Group comparisons were performed using the chi-square or Fisher’s exact test for categorical variables and the Mann–Whitney U test or Student’s t-test for continuous variables, as appropriate. A univariable analysis was conducted to identify factors associated with the presence of dysplasia. The following clinical and endoscopic variables were included in the univariable analysis: age, gender, smoking habit, IBD type, disease duration, family history of CRC, previous dysplasia, previous immunosuppressors or biologic therapies, disease activity, presence of ulcerations, pit pattern, lesion size, location, and lesion morphology. Variables with a *P*-value of <.10 in univariate analysis were considered for inclusion in the multivariable logistic regression model to identify independent predictors. Due to the limited number of outcome events and to reduce the risk of model overfitting, we applied a conservative approach based on the “events-per-variable” rule, including only a maximum of two or three variables in the final model. Selection was guided by clinical relevance and statistical significance in univariate analysis. All analyses were performed using IBM SPSS Statistics for Windows (v.29.0, IBM Corp., Armonk, NY, USA), and a *P*-value of <.05 was considered statistically significant.

## 3. Results

### 3.1. Study population

A total of 98 IBD patients were included in the study. The mean age was 53.2 years (SD ±14.8), and 56 patients (57%) were male. The median IBD duration was 228 months (IQR: 171; range 8-612). More than half of the patients (*N* = 51; 52%) had previously received biologic or immunosuppressive therapies, and 14 (14%) reported a family history of CRC. No patient had a diagnosis of primary sclerosing cholangitis. A low-grade dysplasia in random biopsy samples was previously diagnosed in 14 patients (14%). Demographic and clinical characteristics of patients are summarized in Table 1.

**Table 1.** Clinical and endoscopic characteristics of the study population.

Clinical and endoscopic characteristics	*N* = 98 patients
**Age, years**	
**Median (SD)**	53.2 (±14.8)
**Sex, *n* (%)**	
**Male**	56 (57%)
**Disease duration, months**	
**Median (IQR; range)**	228 (171; 8-612)
**Age at disease onset**	
**Median (IQR; range)**	33 (24.5; 6-74)
**UC, *n* (%)**	76 (78%)
**E2**	24 (32%)
**E3**	52 (68%)
**CD, *n* (%)**	17 (17%)
**L2**	11 (65%)
**L3**	6 (35%)
**IBD-U, *n* (%)**	5 (5%)
**Family history of CRC, *n* (%)**	14 (14%)
**Smoking habit, *n* (%)**	
**Yes**	9 (9%)
**No**	60 (61%)
**Ex smoker**	29 (30%)
**Previous dysplasia, *n* (%)**	14 (14%)
**Previous therapy with IMMs or biologics, *n* (%)**	51 (52%)
**Ongoing therapy with ISS or biologics, *n* (%)**	49 (50%)
**Clinical activity**	
**Partial Mayo score**	
**Median (range)**	0 (0-8)
**Harvey–Bradshaw Index**	
**Median (range)**	1 (1-9)
**Endoscopic activity**	
**Mayo UC score**	
**0, *n* (%)**	24, (32%)
**1, *n* (%)**	13 (17%)
**2, *n* (%)**	14 (18%)
**3, *n* (%)**	25 (33%)
**SES-CD**	
**Median, range**	1 (0-12)
**BBPS**	
**Median, range**	8 (3-9)
**Type of endoscopy, *n* (%)**	
**Virtual chromoendoscopy**	29 (30%)
**Dye chromoendoscopy**	30 (30%)
**White light**	39 (40%)

Abbreviations: IBD-U, IBD-unclassified; CRC, colorectal cancer; ISS, immunosuppressors; BBPS, Boston Bowel Preparation Score.

At the time of endoscopical assessment, the majority of patients (*N* = 62; 63.2%) were in clinical remission or had only mild disease activity (*N* = 20; 20.4%), while 16 patients (16.4%) had moderate disease activity. No patients had severe clinical activity. In terms of treatment, most patients were on mesalamine (*n* = 41), followed by anti-tumor necrosis factor (anti-TNF) agents (*n* = 19), vedolizumab (*n* = 8), ustekinumab (*n* = 10), risankizumab (*n* = 6), and upadacitinib and tofacitinib (*n* = 2 each). A small number of patients were on systemic corticosteroids (*n* = 3) or azathioprine (*n* = 2), and five patients were not receiving any medical therapy at the time of colonoscopy. The clinical and demographic characteristics are summarized in Table 1.

All colonoscopies were performed with high-definition scopes: 39 (40%) with white light, 30 (30%) with dye-based chromoendoscopy, and 29 (30%) using virtual chromoendoscopy (NBI).

**Figure 1.**
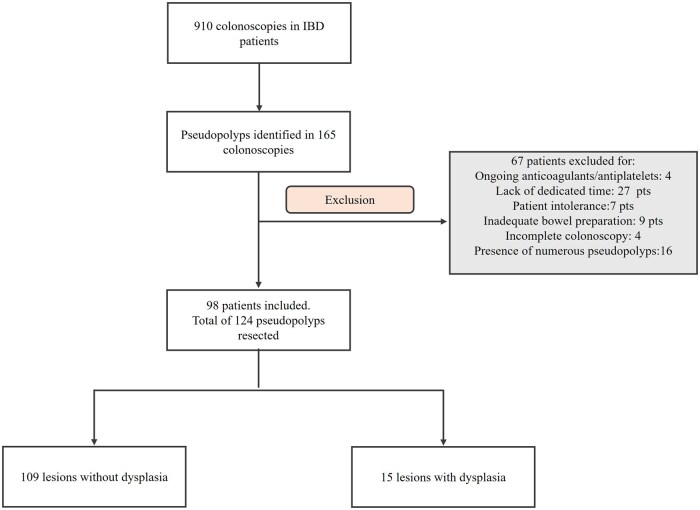
Flow-chart of the study.

Among 910 colonoscopies performed, pseudopolyps were identified in 165 colonoscopies (Figure 1). A total of 124 pseudopolyps were endoscopically resected in 98 patients, including 76 (78%) with UC, 17 (17%) with CD, and five (5%) with IBD-unclassified. The lesions were removed for the following reasons: 22 (18%) were larger than 10 mm, 17 (14%) were isolated lesions, 10 (8%) had a previous diagnosis of dysplasia, five (4%) were in patients with a family history of CRC, and 44 (35%) had a heterogeneous appearance. In total, 114 lesions (92%) were classified as polypoid and 10 (8%) as non-polypoid. The majority were located in the left colon (*n* = 60; 48%), followed by the rectum (*n* = 26; 21%), right colon (*n* = 24; 19%), and transverse colon (*n* = 14; 11%). The median lesion size was 8 mm (IQR: 5; range 6-35).

Surface ulceration was noted in 47 (38%) lesions. The pit pattern was classified as heterogeneous in 22 (18%) cases, while a homogeneous pit pattern was observed in 102 lesions (82%).

All lesions were removed using either standard polypectomy or EMR, with 79 (63.7%) resections performed using cold snare and 45 (36.3%) using hot snare after submucosal injection. Complete resection was achieved in all cases. Immediate post-polypectomy bleeding occurred in nine patients (7.3%), and two patients (1.6%) experienced delayed bleeding. The endoscopic characteristics of the lesion are summarized in Table 2. In cases where pseudopolyps were not resected, the reasons included ongoing anticoagulant or antiplatelet therapy, lack of dedicated time during a diagnostic colonoscopy, patient intolerance, inadequate bowel preparation (defined as a total Boston Bowel Preparation Scale score <6 or any segmental score ≤1), incomplete colonoscopy (defined as failure to reach the cecum), and the presence of numerous pseudopolyps that impaired visualization.

**Table 2.** Endoscopic characteristics of 124 resected pseudopolyps.

Pseudopolyp characteristics	*N* = 124
**Location**	
**Right colon**	24 (19%)
**Transverse colon**	14 (11%)
**Left colon**	60 (48%)
**Rectum**	26 (21%)
**Size, mm, median (IQR; range)**	8 (5; 6-35)
**Surface ulceration, *n* (%)**	47 (38%)
**Number, *n* (%)**	
**Isolated**	74 (60%)
**Multiple**	50 (40%)
**Inflamed surrounding mucosa, *n* (%)**	35 (28%)
**Pit pattern, *n* (%)**	
**Homogeneous**	102 (82%)
**Heterogeneous**	22 (18%)

### 3.2. Histological characteristics and misclassification rate

**Figure 2.**
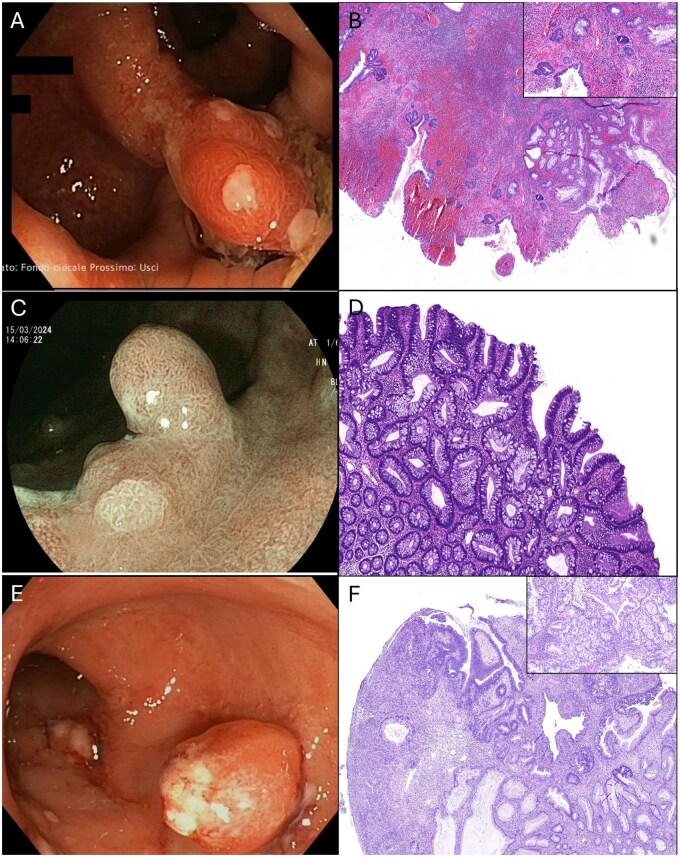
(A) A polypoid lesion (Paris 0-Isp), 13 mm in size, with an ulcerated surface and surrounding hyperemic and edematous mucosa. (B) Inflammatory pseudopolyps with foci of epithelial dysplasia: a polypoid lesion with surface erosion, crypt distortion, glandular hyperplasia, and dense inflammation, together with atypical nuclear features in the mucosal epithelial cells, consistent with foci of epithelial dysplasia (panel) (H&E stain, original magnification ×20). (C) A sessile polypoid lesion (7 mm) with a villous surface pattern and elongated glandular openings, arising from elevated inflamed mucosa. (D) A conventional colorectal adenoma showing clear features of low-grade dysplasia (H&E stain, original magnification ×40). (E) A round polypoid lesion, approximately 10 mm in size, with a smooth surface and areas of superficial ulceration. (F) IBD-associated dysplastic lesion: unequivocal neoplastic alteration of the intestinal epithelium, with superficial ulceration, intramucosal adenocarcinoma/high-grade dysplasia,^26^ and lamina propria infiltration confined to the mucosa (panel), in the setting of IBD-related morphological changes (H&E stain, original magnification ×20).

Among the 124 pseudopolyps resected and histologically analyzed, 15 (12.1%) harbored dysplasia. Notably, eight of these lesions (53.3% of dysplastic cases) were ultimately diagnosed as conventional adenomas: seven with low-grade dysplasia and one as intramucosal adenocarcinoma/high-grade dysplasia.^26^ The remaining lesions with dysplasia included four hyperplastic polyps with low-grade dysplasia and three IBD-associated dysplastic lesions. The endoscopic and histopathological appearance of three representive lesions is shown in Figure 2.

Among the 109 lesions without dysplasia, 76 were classified as inflammatory or hyperplastic polyps related to IBD, while 33 consisted of chronically inflamed tissue that lacked specific histological features for classification as inflammatory/hyperplastic polyps.

All dysplastic changes were confined to the resected lesions, with no evidence of multifocal or synchronous dysplasia. Biopsy samples taken from the adjacent mucosa were negative for dysplasia in all cases, and none of the patients required colectomy following endoscopic resection.

### 3.3. Predictive factors of dysplasia

The predictive factors of dysplasia were analyzed using a logistic regression model. The results of the univariable and multivable analyses for the tested variables are summarized in Table 3. To minimize the risk of overfitting due to the limited number of dysplastic events, only three variables were included in the multivariable logistic regression model: morphology (polypoid vs non-polypoid), presence of ulcerations, and pit pattern (homogeneous vs heterogeneous). In the multivariable analysis, the presence of ulceration was inversely associated with dysplasia (OR = 0.093; 95% CI: 0.01-0.77; *P* = .03), while a heterogeneous pit pattern was positively associated with dysplasia (OR = 4.50; 95% CI: 1.27-15.9; *P* = .02).

**Table 3.** Univariable and multivariable analysis.

Characteristics	Univariable		Multivariable	
	OR (95% CI)	*P*-value	OR (95% CI)	*P*-value
**Age**	1.02 (0.98-1.06)	.24		
**Gender**	2.16 (0.65-7.22)	.19		
**Smoking habit (ref. No smoker)**				
**Active smoker**	0.6 (0.07-5.16)	.64		
**Ex smoker**	0.54 (0.14-2.09)	.38		
**IBD type (ref. CD)**				
**UC**	1.01 (0.20-4.97)	.98		
**U-IBD**	(0.11-20.3)	.76		
**Disease duration**	1.01 (0.99-1.01)	.89		
**Family history of CRC**	1.62 (0.41-6.49)	.51		
**Previous dysplasia**	1.22 (0.31-4.78)	.78		
**Previous ISS/biologics**	2.11 (0.7-6.37)	.18		
**Disease activity (ref. absent/slight)**				
**Moderate**	1.98 (0.43-9.02)	.38		
**Severe**	3.6 (0.99-12.96)	.05		
**Lesion size**	0.95 (0.84-1.08)	.37		
**Location (ref. rectum)**				
**Left colon**	0.3 (0.07-1.22)	.12		
**Transverse colon**	0.7 (0.11-4.18)	.69		
**Right colon**	0.8 (0.19-3.58)	.81		
**Non-polypoid morphology (ref. polypoid)**	3.64 (0.83-15.9)	.08	1.95 (0.29-12.9)	.11
**Ulcerations**	0.09 (0.01-0.77)	.02	0.09 (0.01-0.77)	.03
**Heterogeneous pattern**	3.87 (1.21-12.3)	.02	4.50 (1.27-15.9)	.02

## 4. Discussion

This study was undertaken to evaluate the frequency of dysplasia in pseudopolyps identified and endoscopically resected in IBD patients. Over a nearly 2-year period, pseudopolyps were detected in 18% of colonoscopies, with three-quarters of these lesions being resected and histologically analyzed. Dysplasia was diagnosed in 12% of the cases. These findings challenge the long-standing assumption that pseudopolyps are purely benign post-inflammatory sequelae and support emerging evidence suggesting that such lesions may occasionally harbor or conceal dysplastic foci. Our data not only confirm but also expand on our retrospective study, indicating that up to one-quarter of pseudopolypoid lesions resected during IBD surveillance may contain dysplastic tissue.^15^

Importantly, more than half of the dysplastic lesions in our cohort were histologically classified as conventional adenomas, suggesting that some of these lesions may represent sporadic rather than colitis-associated neoplasia. However, the presence of IBD-associated dysplasia in the remaining lesions lends support to the hypothesis that certain subsets of pseudopolyps may harbor genetic/molecular pathways that contribute directly to carcinogenesis.

Current endoscopic tools, such as NBI and the Kudo classification, have shown limited accuracy in distinguishing between inflammatory and neoplastic lesions in IBD.^21–23^^,^^25^^,^^27^ Although dye-based chromoendoscopy is considered the standard by the SCENIC consensus,^28^^,^^29^ it may still fall short when evaluating morphologically ambiguous lesions, particularly in the presence of architectural distortion due to chronic inflammation. In our study, a heterogeneous pit pattern and the absence of surface ulceration emerged as independent predictors of dysplasia, suggesting that these features could help identify lesions at higher risk of neoplasia during real-time colonoscopy. While the European guidelines currently recommend resecting only “suspicious” lesions,^5^ the data from the present work indicate that even lesions with a benign-appearing pseudopolypoid morphology may still warrant resection, especially if they display a heterogeneous glandular pattern or lack ulceration. This more proactive approach may be justified, given that reliance on visual impression alone resulted in a 12% histological misclassification rate in our cohort.

It should also be acknowledged that similar challenges exist at the histological level, where the reproducibility and definition of pseudopolyps remain subjects of debate. Histological interpretation in IBD is often complicated by overlapping features and interobserver variability, particularly when detecting dysplasia.^30^^,^^31^

Another key takeaway from our study is that no dysplasia was found in biopsies of the surrounding mucosa, suggesting that when dysplasia does occur in pseudopolypoid lesions, it is likely to be focal rather than diffuse This supports the rationale for complete lesion resection rather than partial sampling, which may miss neoplastic foci.

Despite the interesting implications emerging from this analysis, the study has several limitations that must be acknowledged to properly interpret the relevance of the conclusions drawn.

First, this is a single-center study. Although data were collected prospectively, no predefined criteria were established to determine which pseudopolyps should be resected during colonoscopy. The decision to perform resection was made independently by the endoscopist, based on various factors, including bowel preparation, concurrent anticoagulant therapy, patient tolerance to the procedure, and procedure duration. While this undoubtedly represents a selection bias, as the resected lesions may not be representative of the entire spectrum of pseudopolyps occurring in IBD, it also faithfully reflects real-world clinical practice, where the systematic removal of all pseudopolyps during every colonoscopy is not feasible. Second, the relatively small number of dysplastic events limited the statistical power and the scope of the multivariable analysis. Third, we did not include molecular profiling of the resected lesions, which could have further elucidated their malignant potential. Finally, long-term follow-up was not available to assess recurrence or progression to CRC.

Our study also has several strengths. Unlike previously published studies, it is based on a prospectively maintained database, ensuring that all resected polyps were assessed as pseudopolyps by an operator with extensive experience in IBD endoscopy. Furthermore, the complete resection of the lesions, rather than merely performing biopsies, guarantees that we were able to analyze the lesions in their entirety, reducing the risk of missing dysplastic foci, which can occur with biopsy samples.

Our study reinforces the emerging view that a subset of colonic lesions with a pseudopolypoid appearance may harbor dysplasia, highlighting a significant rate of macroscopic misclassification. These findings underscore the diagnostic limitations of current endoscopic assessment in IBD.

The presence of a heterogeneous pit pattern and the absence of ulceration may help identify lesions at higher risk. These findings support the need for a re-evaluation of current surveillance strategies and underscore the potential role of advanced imaging and artificial intelligence-based technologies in improving risk stratification. Future multicenter prospective studies with standardized resection criteria, including molecular analyses, are needed to validate these observations and further refine surveillance strategies.

## Supplementary Material

jjaf196_Supplementary_Data

## Data Availability

The data that support the findings of this study are available from the corresponding author (G.M.), upon reasonable request.
