# Supplementary material for: Frequency of dysplasia in endoscopically resected pseudopolyps in inflammatory bowel diseases
Source: J Crohns Colitis. 2025 Nov 19;19(11):jjaf196. doi: 10.1093/ecco-jcc/jjaf196 (PMC12681356; doi:10.1093/ecco-jcc/jjaf196)
Supplement: jjaf196_Supplementary_Data [file jjaf196_supplementary_data.zip › supplemetary.docx]

Fig. S1

Representative examples of pseudopolypoid lesions in patients with inflammatory bowel disease.
**(A, B)** Homogeneous pseudopolyps evaluated under white-light endoscopy, showing a regular dotted pattern with focal surface ulceration. **(C)** Heterogeneous pseudopolyp displaying peripheral dotted pattern and elongated pit pattern in the central area, assessed using blue-light imaging (BLI). **(D)** Pseudopolyp with a heterogeneous pattern and focal superficial ulceration.
