# Supplementary figures and images for: Frequency of dysplasia in endoscopically resected pseudopolyps in inflammatory bowel diseases
Source: J Crohns Colitis. 2025 Nov 19;19(11):jjaf196. doi: 10.1093/ecco-jcc/jjaf196 (PMC12681356; doi:10.1093/ecco-jcc/jjaf196)

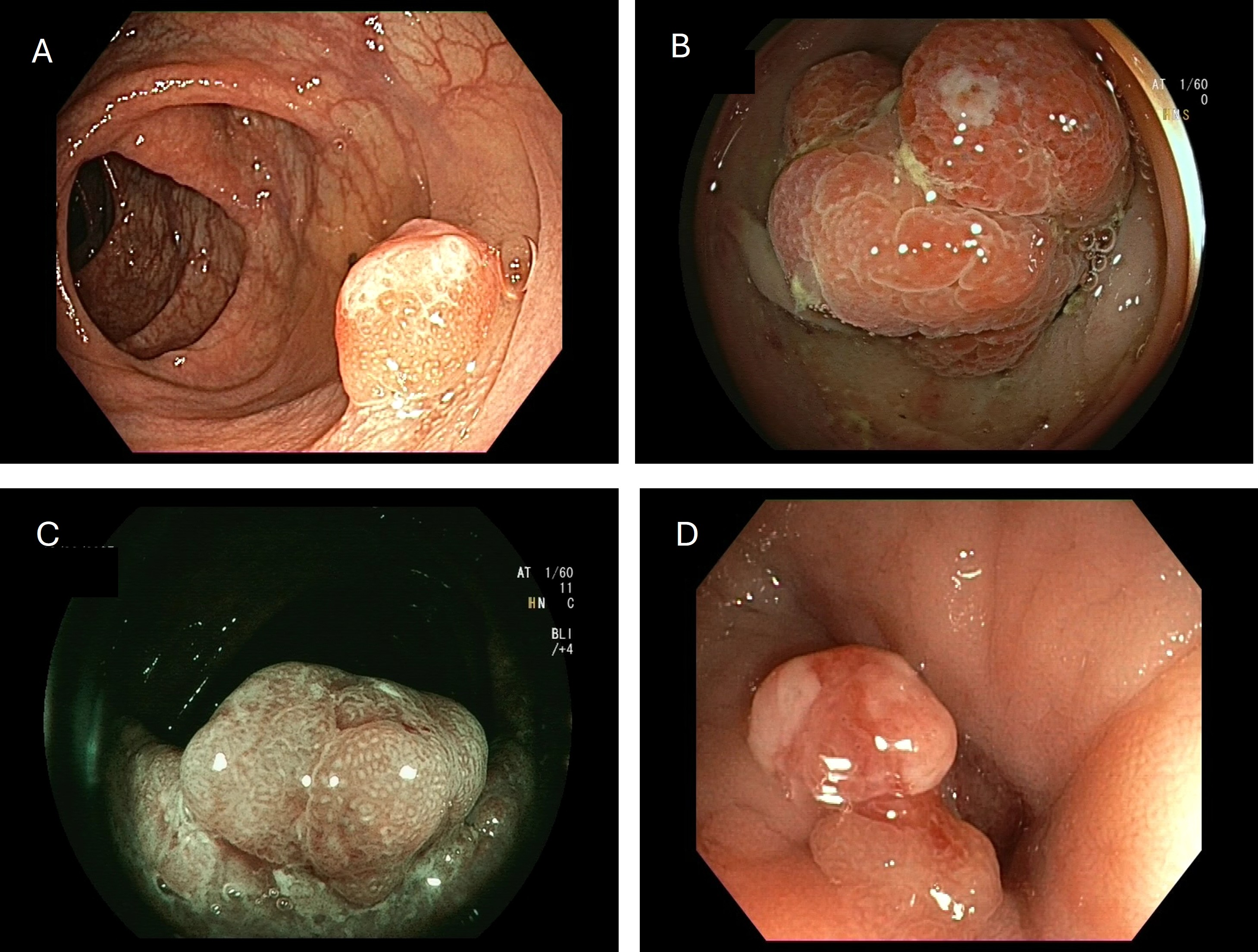

Supplement: jjaf196_Supplementary_Data [file jjaf196_supplementary_data.zip › Fig S1.jpg]
